# Different Effects of Numerical Magnitude on Visual and Proprioceptive Reference Frames

**DOI:** 10.3389/fpsyg.2013.00190

**Published:** 2013-04-17

**Authors:** Elvio Blini, Zaira Cattaneo, Giuseppe Vallar

**Affiliations:** ^1^Department of Psychology, University of Milano-BicoccaMilano, Italy; ^2^Department of Developmental Psychology and Socialization, University of PadovaPadova, Italy; ^3^Brain Connectivity Center, IRCCS MondinoPavia, Italy; ^4^Neuropsychological Laboratory, IRCCS Italian Auxological InstituteMilano, Italy

**Keywords:** visuo-spatial attention, numbers, proprioception, straight ahead tasks, mental number line, ocular movements

## Abstract

This study assessed whether numerical magnitude affects the setting of basic spatial coordinates and reference frames, namely the subjective straight ahead. Three tasks were given to 24 right-handed healthy participants: a proprioceptive and a visuo-proprioceptive task, requiring pointing to the subjective straight ahead, and a visual task, requiring a perceptual judgment about the straight ahead position of a light moving left-to-right, or right-to-left. A control task, requiring the bisection of rods of different lengths, was also given. The four tasks were performed under conditions of passive auditory numerical (i.e., listening to small, “2,” and large, “8,” numbers), and neutral auditory-verbal (“blah”) stimulation. Numerical magnitude modulated the participants’ deviations in the visual straight ahead task, when the movement of the light was from left-to-right, with the small number bringing about a leftward deviation, the large number a rightward deviation. A similar directional modulation was found in the rod bisection task, in line with previous evidence. No effects of numerical magnitude were found on the proprioceptive and visuo-proprioceptive straight ahead tasks. These results suggest that the spatial effects induced by the activation of the mental number line extend to an egocentric frame of reference but only when a portion of horizontal space has to be “actively” explored.

## Introduction

Consistent evidence suggests the existence of strong connections between numerical and spatial representations, with numerical magnitude being represented in the format of a left-to-right oriented horizontal “mental number line” (MNL; Dehaene, 1992), with small numbers lying on the left and large numbers on the right (for reviews, see de Hevia et al., 2008; Umiltà et al., 2009). One of the most interesting consequences of this connection is the finding of orienting effects on spatial attention as a result of magnitude processing, with small numbers inducing a leftward shift of attention, and large numbers a rightward shift (Fischer, 2001; Fischer et al., 2003; de Hevia et al., 2006; Galfano et al., 2006; Casarotti et al., 2007; Nicholls et al., 2008; Cattaneo et al., 2010; Di Luca et al., 2013). For instance, when performing a luminance task participants judged the stimuli darker (with reference to a grayscale placed below or above the stimulus) when a large number was concurrently processed, and brighter when a small number was concurrently processed (Nicholls et al., 2008). Similarly, Casarotti et al. (2007) reported that magnitude processing affects temporal order judgments: in particular, left-sided stimuli are perceived to occur earlier than right-sided stimuli when small digits are concurrently processed, with the opposite pattern emerging when large digits are presented. Furthermore, Fischer et al. (2003) found that small numbers facilitate the detection of stimuli appearing in the left side, whilst large numbers aid the detection of right-sided stimuli, an effect that seems to be automatic (but, see Galfano et al., 2006). Attentional shifts are also preserved in brain-damaged patients showing unilateral biases of attention, namely, unilateral spatial neglect (Bonato et al., 2008; Cattaneo et al., 2012a). In bisection tasks, these effects have been found both in the visual (de Hevia et al., 2006; Cattaneo et al., 2012a), and in the haptic modality, in both sighted (Cattaneo et al., 2012a) and blind (Cattaneo et al., 2010) participants. Simultaneous processing of numerical magnitude affects also spontaneous writing, with healthy participants producing dictated small numbers in the leftward sector of the working space, and large numbers in the rightward sector (Perrone et al., 2010). Furthermore, in a manual-aiming task, participants show shorter response latencies with small numbers, and an effect of congruence between the numerical size and its position in space (i.e., faster movements when aiming leftward to smaller numbers, and rightward to larger numbers, see Ishihara et al., 2006). It is still a matter of debate, however, whether those spatial effects are truly spatial or rather emerge in a response-related stage (see Keus and Schwarz, 2005; Gevers et al., 2006; Daar and Pratt, 2008; Stoianov et al., 2008).

According to the “a theory of magnitude” (ATOM) formulated by Walsh (2003; see also Bueti and Walsh, 2009), numbers, space, time, as well as other quantitative variables likely rely on a general magnitude system, mainly supported by the posterior parietal cortex (see Dehaene et al., 2003; Hubbard et al., 2005). This system, of great evolutionary importance, would operate since birth to provide a common metric for different quantitative phenomena, and would be critical to actively interact with the environment. Accordingly, many studies underline the close relationship between numbers and action planning and execution (Andres et al., 2004; Ishihara et al., 2006; Lindemann et al., 2007; Moretto and di Pellegrino, 2008; Perrone et al., 2010; Chiou et al., 2012; Vicario, 2012). For instance, hand grip movements (i.e., opening or closing) are affected by the size of concurrently presented numbers: with small numbers, hand closing movements are faster and precision gestures are aided, while large numbers accelerate hand opening movements, facilitate power gestures, and lead to a greater maximum hand aperture (Andres et al., 2004; Lindemann et al., 2007; Moretto and di Pellegrino, 2008; Chiou et al., 2012). It is likely that numbers automatically activate motor patterns typically used to interact with objects of different sizes (precision gestures for small objects vs. wider, power gestures for larger objects, see Moretto and di Pellegrino, 2008). Accordingly, Vicario (2012) has recently shown that numerical magnitude affects the choice of using one hand or the other when participants are asked to generate random movements with the fingers: specifically, perceiving small numbers leads to more frequent use of the left-hand fingers, while large numbers lead to a more frequent use of the right-hand fingers (Vicario, 2012). Similarly, when asked to randomly press one of two response keys, individuals tend to choose more often the leftward key when a small digit is presented, and the rightward key when a large digit is presented (Daar and Pratt, 2008). Finally, healthy participants tend to spontaneously generate small numbers when turning the head leftward, and large numbers when turning the head rightward (Loetscher et al., 2008a).

While the effects of numerical magnitude on perception and action have been largely investigated (see above), effects of numerical magnitude on proprioception have been devoted less attention. Proprioception may be defined, in its wider sense, as the awareness of our body in space, with particular reference to the sense of the position and orientation of body parts (Sherrington, 1906; Johnson and Soucacos, 2012). In principle, modulatory effects by numerical magnitude may be also expected in the proprioceptive reference frame. Secondly, proprioception is crucial for the success of an action in the environment: hence, according to the ATOM, the interactive effect between magnitude processing and spatial representations could also involve proprioceptive reference frames. In line with this hypothesis, Eerland et al. (2011) found that quantitative estimates (e.g., the Eiffel Tower’s height) are smaller when healthy participants’ bodies are leaning to the left than to the right, an effect that may be related to the MNL, namely: small numbers would be more available when participants are leaning to the left, with effects of proprioception on quantitative numerical estimates.

Effects of numerical magnitude on proprioceptive coordinate frames, however, have not been investigated so far. This study aimed at verifying whether numerical magnitude may affect the estimation of bodily reference frames. Healthy participants were asked to perform three tasks, which assess egocentric coordinates, the proprioceptive (P), the visuo-proprioceptive (VP), and the visual (V) straight ahead (Redding and Wallace, 1997; Rode et al., 2003; Redding et al., 2005; Fortis et al., 2013). All these tasks have been extensively used across different studies to assess, for instance, body schema distortions in unilateral spatial neglect, sensory-motor transformations (i.e., prismatic adaptation), or the effects of neck-muscles stimulation (Biguer et al., 1988; Taylor and McCloskey, 1991; Farnè et al., 1998; Bartolomeo and Chokron, 1999; Ferber and Karnath, 1999). In particular, the P task was included to investigate purely proprioceptive egocentric shifts (e.g., Farnè et al., 1998; Bartolomeo and Chokron, 1999) possibly induced by magnitude processing. The V task was used to investigate possible horizontal shifts of attention in the external visual space (e.g., Farnè et al., 1998; Ferber and Karnath, 1999). Finally, the VP task was included to investigate whether a visual shift could affect the proprioceptive egocentric space (see Biguer et al., 1988; Taylor and McCloskey, 1991). We chose these tasks in order to allow a direct comparison between our results and the effects previously reported with prismatic adaptation or neck vibratory stimulation. Participants also performed a visual rod bisection (RB) task (in which effects of numerical magnitude have been found before, see Cattaneo et al., 2010, 2012a) as a control condition. Participants performed the four tasks whilst listening to small (“2”) and large (“8”) numbers or to a control auditory sound (“blah”). A passive acoustic stimulation was adopted on the basis of previous evidence indicating that passively perceiving numbers – irrelevant for the task at play – may affect task performance (e.g., Fischer, 2001; Fischer et al., 2003; Cattaneo et al., 2010, 2012a). This would also allow us to directly compare the results of this study with those obtained using line bisection alone (see Cattaneo et al., 2010, 2012a). Moreover, this type of stimulation could be easily administered during all the four tasks employed: we can therefore argue that any possible difference in the results would reflect a different permeability of the specific reference frame to numerical stimulation.

## Materials and Methods

### Participants

Twenty-four healthy volunteers (six males, mean age: 22.6 ± 2.7 years) took part in the experiment, and gave a written consent. All participants were right-handed, and had normal or corrected-to-normal vision. The study was approved by the local Ethical Committee.

### Stimuli and procedure

Participants wore a pair of headphones during all the tasks. During the experimental conditions, a neutral phonological sound (“blah”) or the Italian words for numbers “2” and “8” were auditorily presented. Before starting the experiment, participants were presented with the three types of auditory stimuli, obtained by a vocal synthesizer; no specific information about those stimuli was provided, and participants were said that their task was the same regardless of the stimulus type. Sounds were delivered to a comfortable volume by the means of a portable device. Each auditory stimulus lasted approximately 1 s and was repeated, with the frequency of 1 Hz, until participants’ response. In all tasks, auditory presentation of the stimuli preceded the starting of the task: in particular, each auditory stimulus was repeated five times before the straight ahead or the bisection task started, and continued until the participants’ response was provided. Numbers were presented from 5 s before and throughout the execution of each task, in order to maximize their putative effects on the four tasks. In fact, straight ahead and line bisection tasks require an immediate answer, and the limited response time needed may be insufficient for numbers to exert any detectable effect if numbers presentation is timely triggered with the task.

#### Rod bisection control task

The procedure was similar to that used by Cattaneo et al. (2012a). Participants were seated at a table; the to-be-bisected black wooden rods (30, 35, 40, and 45 cm; 1.5 cm diameter) were placed one at a time on the table in front of them so that the center of each rod was aligned with the body’s mid-sagittal plane of each participant. The rod to bisect was initially covered from sight by a black plastic panel, 30 cm × 60 cm wide. The trial started with the presentation of the auditory stimulus (small number, large number, or “blah”): after five repetitions of the stimulus, the experimenter lifted the panel. Participants were instructed to point to the estimated midpoint with their right index finger. After each trial, the rod was covered again with the panel and replaced by the successive stimulus, namely a rod of different length. Thirty-six trials were given, three for each of the four rod lengths, and for each of the three auditory conditions (small number, large number, “blah”). The order of the rod lengths, and of the auditory cues were randomized. Four practice trials (one for each rod length, with no auditory stimulation) were performed before the experiment, and not included in the analysis. At the start of the experiment, a vertical line (approximately 1 mm wide) was drawn with a pen in the middle of the tip of the participants’ right index finger, as to ease the scoring of data. Deviations from the true midpoint were recorded to the nearest millimeter and converted to a percentage of line length: deviations from the veridical center were converted to signed percentage scores (positive if bisections were to the right, negative if to the left) by subtracting the true half-length of the rod from the measured distance of each setting from the left extremity of it, and then dividing this value by the true half-length and multiplying the quotient by 100 (see Laeng et al., 1996; Rode et al., 2006; Cattaneo et al., 2012a,b).

#### Straight ahead tasks

The paradigms used by Ronchi et al. (2011) and Fortis et al. (2013) were employed (see also Redding and Wallace, 1997). Participants sat at a table with their head aligned with the mid-sagittal plane of their body, and stabilized by a chin-rest attached to the table. A transparent square panel (50 cm side) marked with a goniometer with lines radiating from −90° to +90° was placed on the table, centered on the participants’ mid-sagittal plane. The three tasks and the three auditory cues were presented in counterbalanced order across participants. For the proprioceptive and the visual-proprioceptive tasks participants were asked to perform pointing movements with their right upper limb, as fast and accurate as possible. The participants’ arm was positioned at the center of the panel, with the right-hand resting on the starting location near their body and aligned with the mid-sagittal plane of the body. This served as a starting point for all movements. Each of the three straight ahead tasks included 24 items, eight for each auditory cue (i.e., “2,” “8,” and “blah”); each task was preceded by four practice trials (not included in the analysis) with no auditory stimulation.

(i) *Proprioceptive task* (*P*). Participants, with eyes closed, were instructed to indicate the subjectively estimated position of their body midline on the panel surface. On each trial, the experimenter recorded the deviation of the finger position from the true objective body midline (°, degrees of visual angle).
(ii) *Visual task* (*V*). A red LED was mounted on a pulley (100 cm long, 1.5 cm wide) placed horizontally at the top of a black wooden box (35 cm high, 75 cm long, and 20 cm wide). The box was positioned in a darkened room. The distance between the center of the pulley and the participants was 65 cm.
  The visual test did not involve arm movements: participants were instructed to verbally stop the movement of the LED (approximately 2°/s fast), when its position corresponded to their subjective mid-sagittal plane. A centimeter attached to the pulley on the experimenter’s side allowed for the recording of the deviation of the LED position from the center of the pulley, corresponding to the participants’ physical mid-sagittal plane (*D*, measured in centimeters). Each measurement was then transformed in degrees of visual angle (°) *via* the formula: *D*(°) = [arctan(*D*/65)] × (180/π). The direction of the LED movement was varied and counterbalanced between trials.
(iii) *VP task*. The same pulley-mounted LED box of the visual test was used. Participants performed 24 pointing movements on the panel surface to indicate the downward projected position of the LED. On each trial, the LED was placed in front of the participants’ mid-sagittal plane. The movement of the arm was occluded from vision by a two-layer wooden box (30 cm high, 75 cm wide, and 50 cm deep) and by a black cloth attached from the participant’s neck to the upper surface of the box. Participants were instructed to close their eyes between each trial to allow the experimenter to score their performance.

All participants started with the bisection task; the order of the three following straight ahead tasks was counterbalanced across participants. The entire experiment took approximately 1 h to be completed.

In order to avoid confounding effects of the auditory-verbal stimulation *per se* (Cattaneo et al., 2012b), in all tasks the participants’ deviations in the small and large number conditions were corrected for their baseline (“blah”) deviations, by subtracting the mean score in the baseline condition from the mean score obtained in the small and large number conditions. The signed mean scores were analyzed by repeated-measures analyses of variance (ANOVA), with, as within-subjects factors, numerical condition and rod length (for the RB task), task (the P and VP tasks), and direction of the movement of the LED light (for the V task). Significant main effects and interactions effects were analyzed by Bonferroni corrected multiple comparisons.

## Results

**Figure 1:**
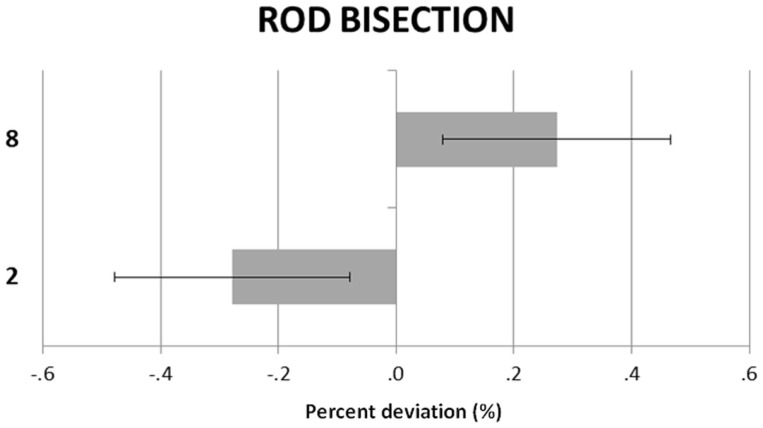
**Rod bisection**. Participants’ mean (SEM) deviations (corrected for the “blah” baseline) in the small (“2”) and large (“8”) number conditions.

Figure 1 shows the participants’ RB deviations in the small and large number conditions. The large number induced a rightward shift and the small number a leftward shift. A repeated-measures ANOVA with numerical condition (small vs. large) and rod length (30, 35, 40, 45 cm) as within-subjects factors showed a significant main effect of condition, *F*(1, 23) = 9.83, *p* = 0.005, $\eta_{\text{p}}^{2}$ = 0.29. Neither the main effect of rod length, *F*(3, 69) = 1.16, *p* = 0.33, $\eta_{\text{p}}^{2}$ = 0.05, nor the rod length by numerical condition interaction, *F*(3, 69) = 1.10, *p* = 0.35, $\eta_{\text{p}}^{2}$ = 0.04, were significant.

**Figure 2:**
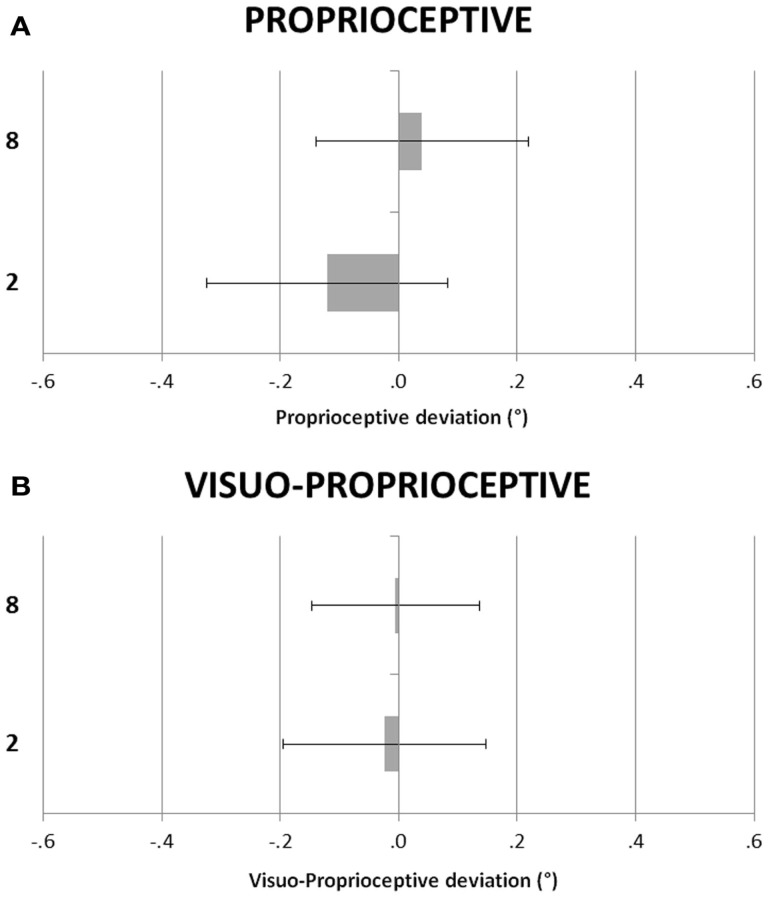
**(A)** Proprioceptive (P) and **(B)** Visuo-Proprioceptive (VP) straight ahead tasks. Participants’ deviations as in Figure 1.

Figure 2 shows the participants’ deviations in the P and VP tasks in the small and large number conditions. A repeated-measures ANOVA was performed with task (V vs. VP) and condition (small vs. large number) as within-subjects factors. The main effects of numerical condition, *F*(1, 23) = 0.94, *p* = 0.34, ${\text{η}}_{\text{p}}^{2}$ = 0.04, and of task, *F*(1, 23) = 0.02, *p* = 0.90, $\eta_{\text{p}}^{2}$ = 0.00, were not significant, as well as the condition by task interaction, *F*(1, 23) = 0.40, *p* = 0.53, $\eta_{\text{p}}^{2}$ = 0.02.

**Figure 3:**
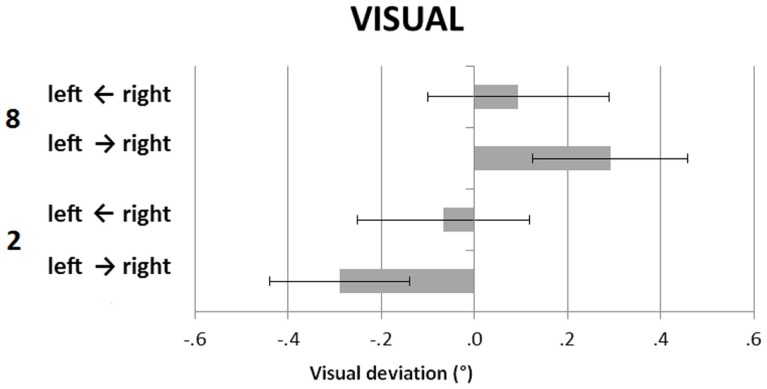
**Visual straight ahead task (V)**. Participants’ mean (SEM) deviations as in Figure 1, and by direction of the movement of the LED light (left-to-right, right-to-left).

Figure 3 shows the participants’ deviations in the V task, in the small and large number conditions, by the direction of the light motion (left-to-right, right-to-left). The light was overall stopped leftward when the small number was presented, and rightward when the large number was presented, with the effect being larger in the left-to-right movement condition of the LED light. A repeated-measures ANOVA with numerical condition (small vs. large) and light direction (light moving rightward vs. light moving leftward) revealed a significant main effect of numerical condition, *F*(1, 23) = 14.67, *p* = 0.001, $\eta_{\text{p}}^{2}$ = 0.39, and no significant main effect of light movement direction, *F*(1, 23) = 0.01, *p* = 0.95, $\eta_{\text{p}}^{2}$ = 0.00. The numerical condition by light movement direction interaction was significant, *F*(1, 23) = 4.45, *p* = 0.046, $\eta_{\text{p}}^{2}$ = 0.16. *Post hoc* analysis (Bonferroni correction applied) showed that during the left-to-right movement of the LED the difference between the small and the large number deviations was significant, *t*(23) = 4.52, *p* = 0.002. Conversely, no difference was found with the right-to-left movement of the light, *t*(23) = 1.08, *p* = 1.00.

## Discussion

The main and novel result of this study is that the modulation exerted by numerical magnitude over spatial judgments extends to the visual straight ahead task, which assesses egocentric coordinates, and is not confined to allocentric frames of reference, such as those assessed by rod and line bisection (Fischer, 2001; de Hevia et al., 2006; Cattaneo et al., 2010, 2012a), and cancellation tasks (Di Luca et al., 2013). Moreover, in line with previous evidence (Cattaneo et al., 2012a), we confirm that processing small and large numbers affects the participants’ performance in visual RB. In both the bisection task and the visual straight ahead task, the individuals’ bias was modulated consistently with the structure of the MNL, namely: listening to small numbers brought about a leftward deviation, listening to large numbers a rightward deviation.

Conversely, the proprioceptive domain seems to be less penetrable by concurrently presented magnitude information. A possible explanation for this may be found in the salience of the elicited horizontal dimension, much greater in the visual bisection and in the visual straight ahead tasks than in the proprioceptive straight ahead tasks. In the visual straight ahead task, and in the visual bisection task, a left-to-right oriented direction is clearly established in external space. In the proprioceptive tasks this is not the case, since these eventually imply a bottom-up vertical direction only, and therefore they may be less sensitive to orienting effects induced by numerical magnitude processing along the horizontal dimension. Accordingly, in the present visual straight ahead task, the effect exerted by numerical magnitude was stronger when the light moved from left-to-right, i.e., in a direction consistent with the order of numbers on the putative MNL. Moreover, the effects of numbers on performance in the visual straight ahead task and in the visual bisection may be more easily mediated by involuntary ocular movements, which are also affected by numerical magnitude (Loetscher et al., 2008b; Knops et al., 2009; Ruiz Fernández et al., 2011; Grade et al., 2013). Finally, we cannot exclude that instructing participants to pay direct attention to numerical magnitude (by for example requiring an explicit judgment on it) may have resulted into numbers also affecting proprioceptive tasks (see Casarotti et al., 2007).

Body posture, a proprioceptive state, may affect quantity estimation tasks (see Eerland et al., 2011). Our data suggest that the reverse does not apply, namely: numerical processing does not appear to affect proprioception, at least as assessed by straight ahead tasks, while modulating the visual straight ahead. From an evolutionary point of view it is plausible to assume that the information regarding magnitude (typically originating from the outside world, see Schmandt-Besserat, 1999; Vallar and Girelli, 2009, for discussion) is not critical for maintaining our own body schema sufficiently stable over time, while the same information would be important to adapt our actions to the environment (see Moretto and di Pellegrino, 2008). Future research may help to shed light on this issue.

## Conflict of Interest Statement

The authors declare that the research was conducted in the absence of any commercial or financial relationships that could be construed as a potential conflict of interest.
